# The Role of Regional Contrast Changes and Asymmetry in Facial Attractiveness Related to Cosmetic Use

**DOI:** 10.3389/fpsyg.2018.02448

**Published:** 2018-12-14

**Authors:** Amanda C. Killian, Sinjini Mitra, Jessie J. Peissig

**Affiliations:** ^1^Department of Psychology, California State University, Fullerton, Fullerton, CA, United States; ^2^Information Systems and Decision Sciences Department, California State University, Fullerton, Fullerton, CA, United States

**Keywords:** face perception, attractiveness, asymmetry, contrast, facial cosmetics, makeup

## Abstract

This study collected behavioral data for testing how regional contrast changes due to the addition of cosmetics would affect attractiveness ratings. In addition, we used an established model of asymmetry to look for a correlation between changes in attractiveness related to the application of cosmetics to specific regions of the face and changes in symmetry. Using this asymmetry model we compared female faces with and without makeup. Specifically, we used a highly controlled set of grayscale faces in which makeup application was standardized to explore these issues from a perceptual perspective. The human data showed that adding upper eye makeup significantly increased attractiveness ratings. In contrast, increases in contrast to the lower eyes and lips did not lead to increases in attractiveness ratings; application of cosmetics to the lower eyes led to a significant decrease in attractiveness. We found that for the makeup condition that led to increased attractiveness, asymmetry did not change significantly when makeup was applied to the female faces. This suggests a role for mechanisms other than symmetry related to increases in attractiveness related to makeup use in females.

## Introduction

Many have attempted to determine what makes a face attractive (Cunningham, 1986; Fink and Penton-Voak, 2002; Baudouin and Tiberghien, 2004; Rhodes, 2006), and facial attractiveness has been known to play a significant role in our social interactions with others (Langlois et al., 2000; Buss, 2008; Schmid et al., 2008; Perilloux et al., 2013). For example, we tend to attribute positive qualities to those perceived as attractive and negative qualities to those perceived as unattractive (Dion et al., 1972). Research also suggests that the desire for attractiveness affects our spending, even during grave financial times (Hill et al., 2012). Hill et al. found that women actually *increased* their financial spending on beauty products like makeup during the latest economic recession. Attractiveness is a trait that may influence behavior across the lifespan, as data indicate that even very young infants discriminate between attractive and unattractive faces (Langlois et al., 1990; Slater et al., 1998). Taken together, these studies suggest a highly significant role for facial attractiveness in our everyday lives.

### Symmetry and Facial Attractiveness

Symmetry has long been associated with beauty in both art and nature, and not surprisingly has been found to be related to facial attractiveness (Rhodes, 2006). Facial asymmetry can be caused either by external factors, such as expression changes, viewing orientation and lighting direction, or by internal factors such as growth, injury, and age-related changes (Liu et al., 2003). The latter is more interesting, being directly related to the individual face structure, whereas the former can be controlled to a large extent and even removed with the help of suitable image normalization. In this study we tested the relationship between facial asymmetry and the perceived attractiveness of a face, particularly in the presence of applied cosmetics. In addition, we explored how applying makeup to different regions of the face affected attractiveness.

**FIGURE 1:**
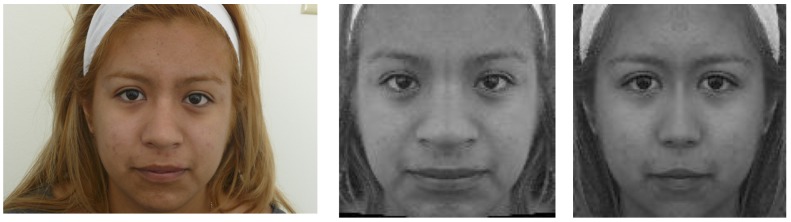
**Left**: original face image from our dataset. **Middle**: a perfectly symmetrical face made of the left half of the original face. **Right**: a perfectly symmetrical face made of the right half of the original face.

Human faces are bilaterally symmetric, however, the two sides of the face are not completely identical. Although it is often difficult to visually perceive the degree of asymmetry in a face, this becomes quite clear in Figure 1 – here a face is re-constructed to create two images by using one side of the face to create a mirror image. It is plainly seen that the two re-constructed faces look different from each other, and both are significantly different in appearance from the original image. This shows that the two halves of a human face are not symmetric. Moreover, the amount of asymmetry in a face varies significantly across individuals, thus it has the potential to be a significant factor in perceptions of attractiveness.

Psychologists have long been interested in the relationship between facial asymmetry and attractiveness. Research conducted over the past two decades has shown that facial symmetry is a desirable quality for potential mates – the more symmetrical the face the better (Grammer and Thornhill, 1994; Gangetsad and Thronhill, 1997; Rhodes, 2006). Indeed, some researchers go further to say that symmetry in the body (excluding the head; Tovée et al., 2000), or between other bodily features, such as hands, is also preferred. The usual assumption is that bodily symmetry is a proxy for good health (Penton-Voak et al., 2001; Fink et al., 2006). Symmetry suggests orderly development in the womb and during childhood, and thus, according to these hypotheses, captures a range of desirable things from good genes to infection-resistance. In support of the idea that symmetry may be used as a cue to health in mate choice are studies that show that symmetry is related to other mate cues, such as body odor (Rikowski and Grammer, 1999) and voice (Hughes et al., 2002). Scheib et al. (1999) conducted a study with 79 female students which found that a relationship exists between women’s attractive ratings of men’s faces and symmetry. Korichi et al. (2011) observed that the females in their study who appeared to use makeup more as a camouflage, or to blend in, exhibited greater asymmetry in the lower part of the face compared to those who applied cosmetics to enhance their attractiveness. This suggests a possible relationship between makeup use and asymmetry.

Other studies have explored facial symmetry in relation to environmental conditions (Little et al., 2007; Dixson et al., 2017). These studies have found that for populations that experience harsher conditions, there is a stronger preference for symmetry. In particular, Dixson et al. (2017) found that when comparing Melanesian participants from three different islands, those that were exposed to higher rates of malaria exhibited a higher preference for symmetrical faces. This preference for symmetry also extends to facial ornamentation, such as face paints (Cárdenas and Harris, 2006). These studies provide more evidence of a relationship between perceptions of health and the level of symmetry.

Alternatively, people may find facial symmetry attractive for other reasons. There is very little evidence that facial symmetry is related to an individual’s objectively measured health, even in cases in which more symmetrical individuals are perceived as healthier (Rhodes et al., 2001). This suggests that even though people judge symmetrical individuals as more attractive and healthy, underlying health may be only moderately associated with symmetry or not associated at all. So, if not related to health cues, why do people rate symmetrical faces as more attractive? One hypothesis suggests that people may have an overall preference for symmetry in visual perception merely as a by-product of the structure of the visual system (Enquist and Arak, 1994; Johnstone, 1994; Enquist and Johnstone, 1997). This perceptual preference is then applied to judgments of facial attractiveness. Regardless of whether the preference for symmetry is the result of an evolved mate cue or a perceptual system bias, the question remains as to whether facial attractiveness manipulations, such as wearing makeup, might affect facial symmetry. This could explain at least one perceptual route by which the application of cosmetics could increase attractiveness.

### Contrast and Facial Attractiveness

Another factor that may be important for perceptions of facial attractiveness is contrast. Russell (2009) found that luminance played a key role in perceiving a face as male or female. By enhancing or decreasing the regional luminance differences in an androgynous face he was able to shift people’s perception of the face to either more feminine or more masculine (The Illusion of Sex^1^). Specifically, he manipulated the contrast difference between both the mouth and eye regions and the immediately surrounding skin of both these areas. Russell found that making a face appear more feminine, by increasing the contrast in both the mouth and eye regions, led to an increased perception of attractiveness for female faces (Russell, 2009). In addition, he found that females tend to apply cosmetics to these same facial regions, perhaps enhancing sexual dimorphism and attractiveness. Other studies have found that lip color (perhaps in addition to contrast differences) plays a role in attractiveness, perhaps as a cue of health (Stephen and McKeegan, 2010). These findings, along with others, support the idea that perceptions of beauty are not merely arbitrary cultural inventions, but rather are consistent across race and culture (Fink and Neave, 2005); instead, perceptions of beauty may be related to perceptual biases, in this case a bias for increased contrast.

In another study exploring facial contrast, Jones et al. (2015) found that the contrast in the eye region was significantly related to sexual dimorphism. In particular, they found that females have less contrast than males in the eyebrows and more contrast than males in the eye region. Jones et al. also showed that females use makeup to exaggerate these dimorphic differences in the eye region. The effects for the mouth were significantly smaller, suggesting the eye region may play a bigger role in the contrast changes that lead to increased attractiveness.

In this study, we extend Russell’s findings (Russell, 2003, 2009) to better understand the underlying mechanism involved in makeup manipulations of attractiveness. To do this we collected our own face stimulus set by adding contrast using controlled application of makeup. This created more equivalent increases in contrast for each application type, and eliminated possible biases in how individuals apply their own makeup. Although these biases for self-application are interesting to explore, our intention was to start with a more controlled set of stimuli first. We then used these facial stimuli to compare specific regions of increased contrast (lips, upper eyes region, and lower eye region); it is unclear in Russell’s original studies whether the eye region or the lip region contributes more to this effect, or if both regions contribute equally. The Jones et al. (2015) suggests a bigger role for the eye region in the attractiveness increases related to contrast changes that warrants additional testing. Additionally, we wanted to test whether some types of contrast increases, even within the eye region, may actually decrease attractiveness, to eliminate the possibility that any contrast increase will increase attractiveness. Previous research has emphasized the importance of eyes in perceptions of attractiveness (Kampe et al., 2001) and other perceptual tasks (Keil, 2009), supporting the proposal that people tend to focus on the eyes.

### The Role of Cosmetics

Applying artificial makeup is an example of a cosmetic alteration that can change the perceived appearance of a face and is known to enhance its attractiveness as well. In contrast to surgical alterations (such as those resulting from plastic surgery) which are costly and permanent, makeup provides non-permanent alterations that tend to be simple and cost-efficient, and thus are more common. Despite this, cosmetic use has the potential to substantially change a person’s appearance by changing facial contrast, skin quality and color. Law Smith et al. (2006) found that the faces of women wearing makeup were rated more positively on a number of measures, including attractiveness, health, and femininity. These are some of the same qualities affected by symmetry and contrast.

Other studies have reported significant increases in attractiveness when female faces are shown with cosmetics (Cash et al., 1989; Mulhern et al., 2003; Nash et al., 2006; Guéguen and Jacob, 2011). Cash et al. (1989) found that college women rated their own self-image as more positive when they were wearing cosmetics. They also found that men rated the face images with makeup as more attractive than those without makeup. Women rating the same faces showed no difference between the faces with and without makeup. Mulhern et al. (2003) compared different types of makeup application, as well as full makeup vs. no makeup. They compared full makeup and no makeup to foundation only, eye makeup only, and lip makeup only. They found sex differences in the perception of different types of makeup application. Mulhern et al. reported that both males and females rated full makeup as more attractive than no makeup on the same faces. Looking at the sexes separately, they found that females rated eye makeup as the biggest contributor to facial attractiveness. Males, however, rated both foundation and eye makeup as contributing similarly to attractiveness. Both males and females rated lipstick as not contributing significantly to attractiveness. This study suggests that there may be differences between different regions of the face and the effect cosmetics have on attractiveness.

The general finding that makeup enhances female attractiveness applies outside the laboratory as well. For example, Guéguen and Jacob (2011) found that men tipped more generously when their female waitress was wearing makeup versus when she was not. Jones and Kramer (2015) compared the level of increase in attractiveness related to makeup, and found that the increase in attractiveness was relatively small when compared to the differences in attractiveness found between individuals. However, even if the effects of makeup on attractiveness are relatively small, a small increase may be worth the effort, especially considering the numerous advantages conferred to more attractive individuals (van Leeuwan and Macrae, 2004). In addition, the benefits of makeup may go well beyond simple increases in attractiveness. Numerous studies have shown that the same faces shown with makeup were perceived as more prestigious and dominant (Mileva et al., 2016), as having jobs with higher status (Richetin et al., 2004; Nash et al., 2006), and as more competent (Klatt et al., 2016). Support for this can be found in the high level of spending found for cosmetics; a Groupon survey showed that women in the United States spend on average $28 every month on makeup alone (Haynes, 2017).

### Face Database

In this study, we tested a face database consisting of 30 individual females. They were recruited from the human participant research pool of the Psychology Department at California State University Fullerton. The participants were given course credit for participation and gave permission for their images to be used in experiments.

**FIGURE 2:**
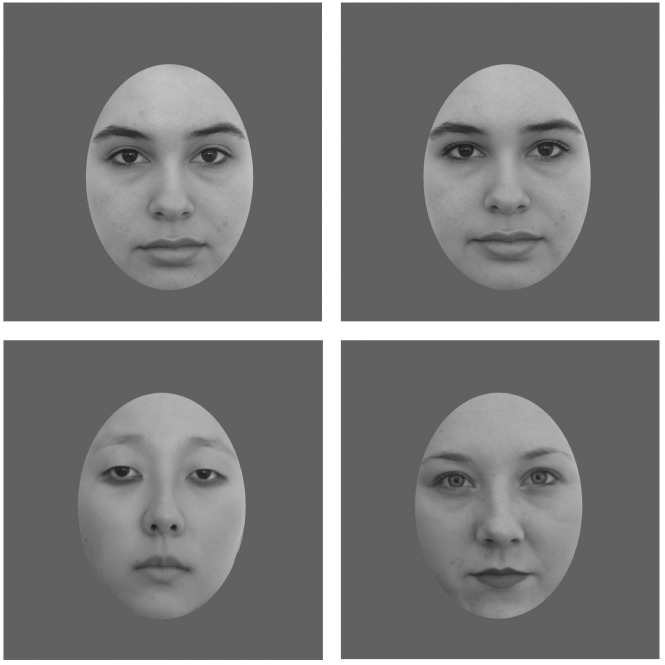
**Top Left**: example of original face with no-makeup. **Top Right**: example of face with upper eye makeup. **Bottom Left**: Example of face with under eye makeup. **Bottom Right**: Example of face with lip makeup.

We chose to use a more ecologically valid method of applying cosmetics directly to the faces (rather than a computer manipulation of contrast or morphing). We had an experimenter apply the makeup so that the application would be consistent across this particular set of faces. The faces were collected using a standardized method, holding constant the lighting and distance of the camera. Participants were required to have no previously applied makeup on their faces prior to adding cosmetics and their hair was pulled back from the face to ensure a clear view of the entire face. The same colors of makeup were applied to each participant by an experimenter to one of three areas of interest: lipstick applied to mouth region (lips); eye shadow and mascara applied to the upper eye region; eye shadow applied to the lower eye region (Figure 2). The specific makeup application regions were chosen to allow us to test upper eye makeup and lip makeup separately. We also included the lower eye makeup as a comparison condition, to show that adding eye makeup to any part of the eye region is not sufficient to lead to increases in attractiveness. For each individual, we only used a single makeup application, i.e., each face only had lipstick, upper eye makeup, or lower eye makeup applied. This was done to prevent issues with changes in skin quality and discoloration caused by makeup removal. In addition, some types of makeup are particularly difficult to remove, such as mascara. We were concerned that some makeup remnants would remain even after attempted removal, which would not allow us to fully test a single makeup application type. Using Photoshop 4.0, each face had a uniform mask applied to help eliminate confounding features (e.g., hair; see Figure 2). The faces were shown in grayscale, to control for the contribution of color to attractiveness ratings and to allow us to focus specifically on how changes in contrast relate to attractiveness (Russell, 2003, 2009). Once the mask was applied to each image and the image converted to grayscale, the contrast increase between the same faces without makeup and with applied makeup was calculated. To calculate the contrast increase caused by the application of cosmetics, we used an adapted version of Michelson contrast formula to produce a facial contrast value (*C*_F_), as used by Russell (2003, 2009). The mean *C*_F_ values were similar across all three regional applications of makeup (lips 0.115, upper eyelids and upper eyelashes 0.121, under eyes 0.133). A one-way ANOVA showed that there was no significant difference among these *C*_F_ values, *F*(2,29) = 0.126, *p* > 0.05. Sample images from our database are shown in Figure 2.

A summary of our database appears in Table 1. We had a total of 30 individuals in our database. Each person had one image with no makeup and one image with makeup (either lips, upper eyes or under eyes). Thus, there were a total of 60 images, 30 of them having no makeup and 30 with one of the three types of makeup applied.

**Table 1:** The number of faces under each contrast condition in the experiment. Each face set consisted of 10 individual faces shown with and without makeup for a total of 60 stimuli.

		Makeup face database design	
		Original (no makeup)	Changed (with makeup)
Face set A	Lips	10	10
Face set B	Upper Eyes	10	10
Face set C	Lower Eyes	10	10

Each image was aligned and normalized according to the steps mentioned in Liu et al. (2001). This was to establish a common coordinate system such that quantified asymmetry measures could be computed and compared meaningfully across all the different faces.

## Materials and Methods

Our experiment consisted of three stages:

(1) Computing quantified asymmetry measures from these faces to study how asymmetry varies with the application of different types of artificial makeup.
(2) Human participant rating of these faces on attractiveness, for both the original faces (no makeup) and the same faces with makeup applied to one region.
(3) Comparing attractiveness ratings with asymmetry metrics to study the relationship between these two metrics, both with and without makeup.

In this study we explored whether changes in the perception of facial attractiveness due to a standardized application of cosmetics use were related to changing the symmetry of the face. It is possible that makeup is able to mask or offset asymmetries in the face, serving as at least one possible contributing factor in the specific mechanism for how makeup increases attractiveness.

### Facial Asymmetry Measurements

Once a face midline is determined via alignment and normalization, each point in the normalized face image has a unique corresponding point on the other side of the image (given an even number of columns in the image). We used a coordinate system in a normalized face image, with the X-axis being perpendicular to the face midline and the Y-axis coinciding with the face midline. “I” denotes a normalized face, and “I”’ denotes its vertically reflected image. The asymmetry measurement is then defined as the pixel intensity variation between the two halves of the face (in a similar way as introduced in Liu et al., 2001):

Density Difference (D-face):

D(x,y)=I(x,y)−I′(x,y)

**FIGURE 3:**
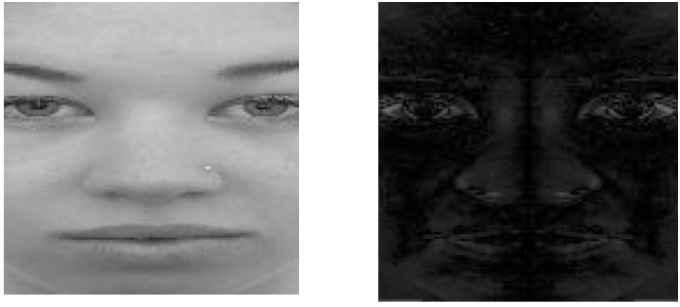
**Left**: Original normalized face image, **Right**: D-face.

Figure 3 displays one normalized face from our database and its respective D-face. Higher D-face values indicate that a face is more asymmetrical; conversely, lower D-face values indicate that a face is less asymmetrical. By construction, the left and right halves of D-face are opposite. Therefore, half of D-face contains all the needed information for the purpose of identification. From now on, we refer to these half faces as D-face. In Table 2, we define two projections of D-face – *D*_x_, *D*_y_. Note that the *D*_x_ features span along the x-axis on the coordinate system, thus from the middle of the face to the side of the face. The *D*_y_ features, on the other hand, span along the y-axis of the coordinate system, thus from the forehead to the chin. Each dimension is called a feature, so we have two related but differently quantified measures representing facial asymmetry. These features were computed for each image in our database. The means for D-face calculations are show in Table 3.

**Table 2:** The different asymmetry features based on D-face.

Asymmetry features	Description	Dimensions (number of features)
D_x_	Column mean of *D-face* on X-axis	64
D_y_	Row mean of *D-face* on Y-axis	128

**Table 3:** Average*D*-face values for the 30 different face stimuli for original face and faces with different artificial cosmetics applied.

	Average D-face values without make up	Average D-face values with make up	Statistical test *P*-value
Upper eyes (*n* = 10)	25.99 (9.006)	24.86 (6.576)	0.287
Under eyes (*n* = 10)	26.39 (3.57)	28.09 (3.56)	0.0471
Lips (*n* = 10)	25.87 (6.66)	24.54 (7.85)	0.1331

*The values in the parentheses denote the standard deviations across stimuli.*

We would like to note here that there are several other ways to measure facial asymmetry, for example methods based on edge orientations (Liu and Palmer, 2003). Thornhill and Gangestad (1994) computed asymmetry based on differences in the lengths and widths of certain body parts (not just face) like ears, elbows, feet, and observed that facial attractiveness can be predicted by the symmetry in men’s body traits. Scheib et al. (1999) computed facial asymmetry of male faces from digitized photos based on deviations of landmark points on bilateral locations of the face (like eye corners, cheekbones, outer edges of the nose, etc., from a line, in both vertical and horizontal directions) that were later compared with human ratings. We chose the D-face measure in this experiment because it captures the asymmetry of the entire face without focusing on any particular region to explore its relationship with attractiveness under different makeup applications for female subjects. We felt this was particularly important in the initial explorations of this relationship.

### Facial Attractiveness Ratings

Thirty-four human participants (different from those used to create the face database) were recruited using the CSUF psychology department research participant pool. Participants were either given course credit or paid $7 for their participation. Participants rated the 60 facial stimuli (30 faces shown both with and without makeup). The images were completely randomized within a single block. Participants rated the faces using a 7-point Likert-like scale on which “1” indicated “very unattractive” and “7” indicated “very attractive.” Trials started with a fixation cross shown for 500 ms, followed by a face image. The face image was shown until the participant had responded or 5000 ms had elapsed.

### Analyses

**FIGURE 4:**
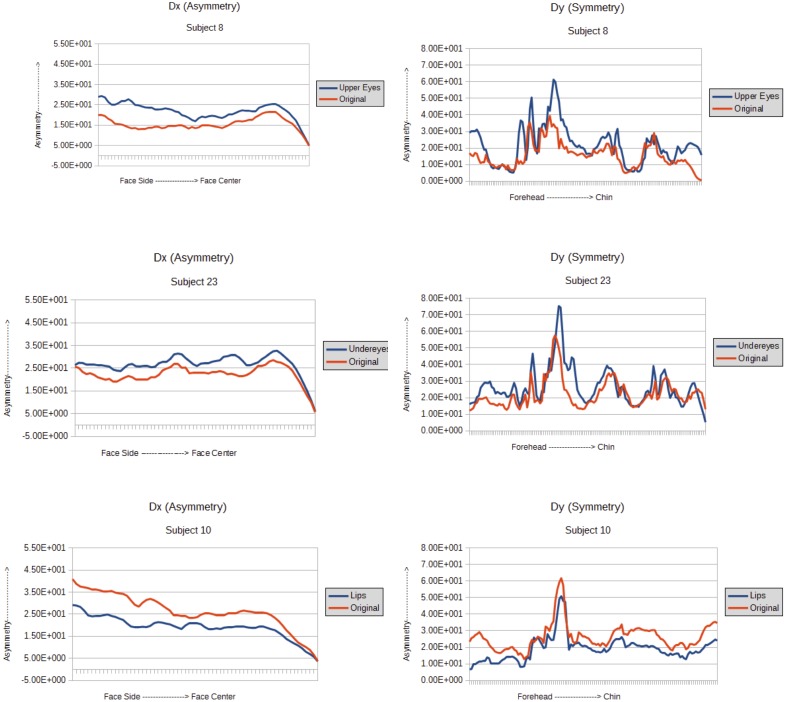
**Left**: *D*_x_ values from 3 subjects with the three different types of makeup – upper eyes, under eyes, and lips. **Right**: *D*_y_ values from 3 subjects with under eye makeup.

Here we summarize the results from our experiments and analyses regarding the relationship between facial asymmetry and attractiveness, both with and without the application of cosmetics. Figure 4 shows average asymmetry values for a randomly selected subset of 10 individual subjects in the database, one value for the original face with no makeup and one value for faces with each of the three different types of applied makeup. The *D*_x_ graph clearly shows that asymmetry of the face gradually decreases from the side of the face towards the center, on average. For *D*_y_, on the other hand, the highest amount of asymmetry seems to be near the nose bridge area and then it decreases as one moves along towards the forehead and also towards the chin. Comparing the asymmetry values across the two dimensions, we find that the nasal area has the highest value, thus indicating that the area around the nose has maximum asymmetry. This is consistent with prior studies that observed that the asymmetry of the nose bridge is significant and it plays an important role in face recognition (Liu and Palmer, 2003).

It is clear that the overall pattern of asymmetry in both dimensions remain unchanged as a result of makeup application, regardless of whether it was applied to the upper eyes, lower eyes, or lips. Another thing that we observed is that application of makeup on the lips decreases asymmetry whereas the makeup in the eye region increases asymmetry. Moreover, the nose area has the highest amount of asymmetry in the whole face in all cases (denoted by the peak in *D*_y_ graphs).

Table 3 summarizes the average *D* values for the individuals in the face database without makeup and with each of the three types of makeup application. The results show that mean asymmetry is slightly higher for original faces than faces with makeup applied on the upper eyes and on the lips, whereas asymmetry increased with the application of makeup under the eyes. Among the three types of makeup studied here, only the under eye changed the asymmetry of the face significantly, as demonstrated by the result from a *t*-test [*t*(9) = -1.87; *p* = 0.0471] whereas the other types of makeup did not (*p*-values are 0.287 and 0.1331 for makeup applied to upper eyes and lips respectively). The effect size (Cohen’s d) for the significant *t*-test for the difference of asymmetry between faces with no makeup and makeup under the eyes was calculated as 0.65 which shows a moderate effect. We thus conclude that cosmetics applied to the upper eye region and lips did not significantly alter the average asymmetry of the face, whereas under eye makeup led to a significant increase in asymmetry.

### Human Participant Data

We analyzed attractiveness ratings using an ANOVA with a two [contrast level: original (i.e., no makeup) vs. changed (with makeup added)] x three (region: upper eyes, under eyes, and lips) design to test for effects in responding. The dependent variable was the attractiveness rating. An overall significant main effect of region was found in attractiveness ratings, *F*(2,66) = 39.67, *p* < 0.0001, η^2^ = 0.0135. A significant main effect of contrast level was also found, *F*(1,33) = 12.15, *p* < 0.001, η^2^ = 0.006. The interaction between contrast level and regions was also significant, *F*(2,66) = 26.16, *p* < 0.0001, η^2^ = 0.024. To explore this interaction further, we used planned contrasts to test for differences between the same faces with no cosmetics applied (original faces) and the different regional makeup applications. Faces with makeup applied to the upper eyes (*M* = 3.89, *SE* = 0.041) were rated as significantly *more* attractive than original faces (*M* = 3.76, *SE* = 0.041), *t*(66) = -2.15, *p* = 0.04. Faces with makeup applied under eyes (*M* = 2.93, *SE* = 0.041) were rated as significantly *less* attractive than original faces (*M* = 3.39, *SE* = 0.041), *t*(66) = -7.94, *p* < 0.001. Finally, for faces with lip makeup applied there was no significant difference between faces with lip makeup (*M* = 3.115, *SE* = 0.041) and original faces (*M* = 3.19, *SE* = 0.041), *t*(66) = -1.43, *p* > 0.05.

**FIGURE 5:**
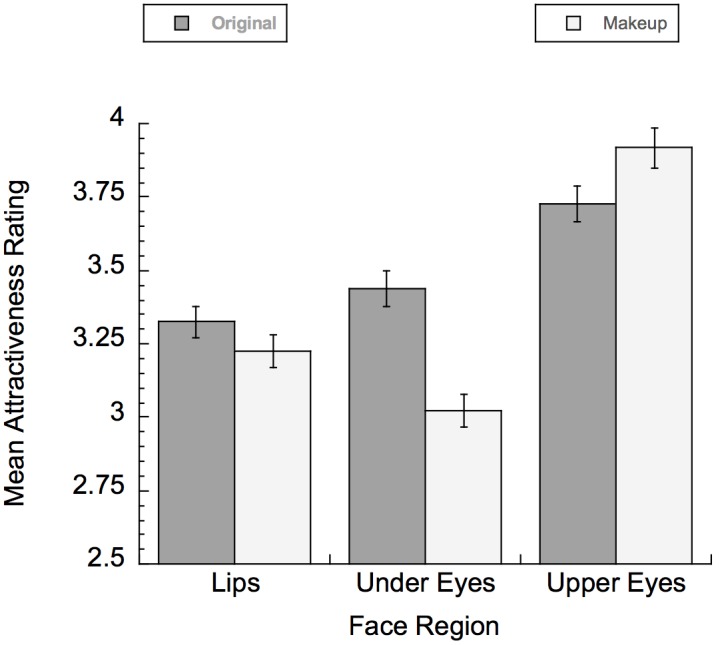
Mean attractiveness ratings for faces with makeup applied to the lips, under eye, or upper eye regions.

The findings suggest that for these images of female faces, an increase in attractiveness ratings was found only for the application of upper eye cosmetics. In contrast, female faces with makeup applied to the under eye region were found to be significantly less attractive than the same female faces with no makeup. Female faces with only added makeup to the lip region were not rated different from the same faces without makeup (see Figure 5).

### Comparing Asymmetry Values With Attractiveness Ratings

As reported earlier, application of artificial cosmetics to the upper eyes and lips reduce facial asymmetry (although not statistically significantly) whereas under eyes makeup increases facial asymmetry. Given the perceived notion that higher asymmetry is associated with lower attractiveness, based on these measures we could conclude that upper eye and lip makeup slightly increased facial attractiveness (as measured by symmetry measures) while make up under the eyes decreased attractiveness.

**FIGURE 6:**
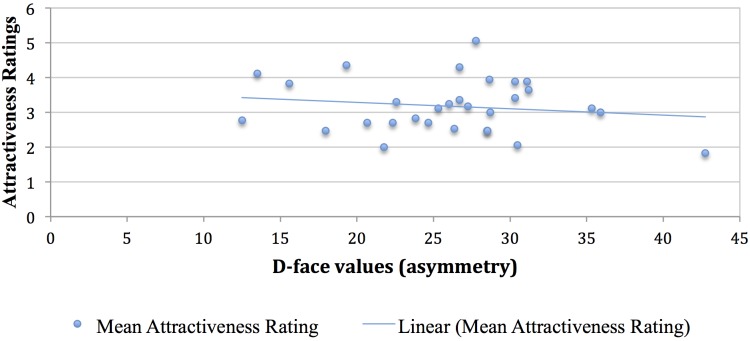
Relationship between facial asymmetry values (D-face) and mean attractiveness ratings for 30 individuals in the face database with no makeup.

**FIGURE 7:**
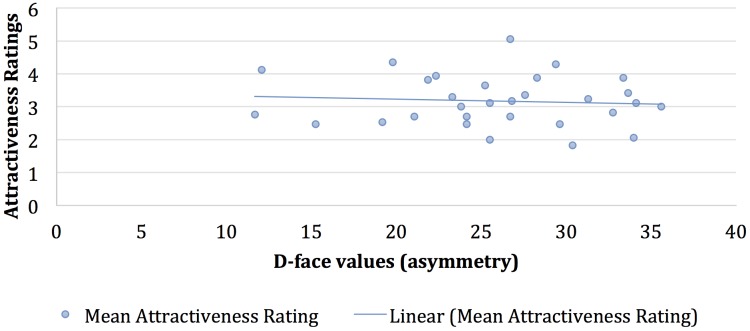
Relationship between facial asymmetry values (D-face) and mean attractiveness ratings for the same 30 individuals in the face database with cosmetics applied.

For the original faces without any cosmetics, we computed the average *D*-face values and the mean attractiveness ratings in order to obtain a baseline, and a negative correlation of -0.16 was observed. Although this conforms to our prediction, note that the association is weak, and not statistically significant (*p* > 0.05). The scatterplot (see Figure 6) shows this slightly negative trend in the association of facial asymmetry values with attractiveness ratings for the faces without makeup. With cosmetic makeup applied, the correlation between the average *D*-face values and the mean attractiveness ratings decreased to -0.08, also statistically not significant (*p* > 0.05). Figure 7 shows the scatterplot for the faces with makeup applied. There is almost no difference in the nature of the relationship between average *D*-face values and the mean attractiveness ratings, for faces with and without any makeup, and they both depicted a slightly negative linear association.

## Conclusion and Discussion

Facial asymmetry is a strong biometric, with the potential of explaining how makeup affects attractiveness. The relationship between facial symmetry and facial attractiveness is well-established. Thus, it seems possible that cosmetics may at least in part increase attractiveness by decreasing asymmetry. In this paper we explored this via a study of quantitative asymmetry metrics and human behavioral attractiveness ratings.

Our results suggest that for human participants, the addition of cosmetics to some regions increased attractiveness ratings (upper eye makeup), whereas other cosmetics applications decreased attractiveness ratings (lower eye makeup), and still other types of cosmetics applications did not change perceived attractiveness ratings (lips). These data suggest that changes in contrast due to cosmetics added to the mouth and eye regions separately are not equivalent. This refines the results reported by Russell (2003, 2009), which only looked at changes to the mouth and eyes in combination, never separately. Our findings are also consistent with the results reported by Mulhern et al. (2003) and Jones et al. (2015). Mulhern et al. found that people reported that applying eye makeup (upper eyes in their case) significantly increased attractiveness. In support of this, Jones et al. found that the eye region was important for sexual dimorphism and the lip region much less so.

In addition, our behavioral data show that not all contrast changes affect attractiveness ratings equally. We found that even when looking at just the eye region, applying makeup to the upper eyes increased attractiveness, and applying makeup to the lower eyes decreased attractiveness. There are many possible explanations for why adding makeup to the upper eye region increases attractiveness. One possibility is that making the area around the eyelashes look darker increases the apparent length of the eyelashes. Eyelashes may be related to levels of estrogen, and thus provide a sign of fertility. For example, some research suggests that hair growth is related to estrogen in women (Hinsz et al., 2001), which may explain why longer hair in females may be rated as more attractive (it can be used as a cue indicating fertility). A role for estrogen in attractiveness seems possible; it has been reported that estrogen is highly correlated with other feminine traits such as hip to waist ratio and facial spacing (Buss, 2008). It is possible that this phenomenon may apply to eyelashes as well. If longer eyelashes are a sign of high fertility, then any increases to the actual length of the eyelashes, or even the illusion of increased length, will significantly increase perceived attractiveness. This phenomenon could explain the popularity of mascara, false eyelashes, eyelash extensions, and drugs that increase eyelash length (e.g., LATISSE^®^). It is interesting to note that across cultures the use of black or brown makeup to darken eyelashes is relatively universal, whereas other colors are not commonly used. This is not true of other types of makeup, in which color choices are much more variable. On the other hand, darkening the under eye region may have indicated poor health, lack of sleep, or disease (Roh and Chung, 2009; Jones et al., 2016). Applying dark cosmetics under the eyes likely gave the appearance of a less healthy and thus less attractive look. We included this condition as a comparison, to show that the precise location of the increased contrast is critical.

There are several other possible mechanisms that may underlie how cosmetics increase attractiveness. Morikawa et al. (2015) found that adding eyeshadow to the upper eyes gave the illusion that the eyes were bigger. Bigger eyes are often perceived as more attractive. Thus, increasing perceived eye size is another possible mechanism for how upper eye makeup in particular increases attractiveness. In another study, Jones et al. (2018) found that cosmetics change the spatial frequencies in female faces. Similar to the findings of Morikawa et al., these changes in spatial frequency made facial features look bigger. Consequently, it may be that any changes that make facial features, particularly eyes, look bigger may increase attractiveness.

Adding contrast to the lip region in the form of lipstick did not significantly increase attractiveness in female faces. Previous research has shown that adding color and contrast to the lip region increases the perception of good health and attractiveness (Stephen and McKeegan, 2010). One main difference between our study and these previous studies is the presentation of our stimuli in grayscale. We specifically chose to use grayscale images, due to studies showing the importance of contrast changes and attractiveness (Russell, 2003, 2009). Color may play a larger role in the increased attractiveness for the application of lipstick, and other types of cosmetics such as blush. It may also be that lipstick does not significantly increase attractiveness. Mulhern et al. (2003) found that both males and females reported that lipstick did not contribute significantly to increased attractiveness. Additional studies will be necessary to better determine under what conditions adding makeup to lips changes attractiveness ratings.

One possible problem with our behavioral data is that we did not equate for overall attractiveness of individuals with no makeup across the different conditions. We were comparing the same individuals with and without makeup, so in one sense any differences should not matter. However, it is possible that the perception of how makeup affects attractiveness might differ depending on whether an individual is thought to be generally attractive or unattractive. For example, for individuals that are already perceived as very attractive, makeup application may create a larger effect (makes attractive women appear even more attractive) or a smaller effect (they are already attractive so there is not as much room for an increase in attractiveness). A similar type of effect might be found for individuals rated as very unattractive. Jones and Kramer (2016) tested how the application of cosmetics change the perception of individuals with differing levels of attractiveness prior to adding makeup. They found that those women that were initially more attractive showed a smaller increase in attractiveness when cosmetics were worn compared to those individuals that were initially rated as less attractive. This suggests that the effects that we found would actually be smaller for the slightly more attractive upper eye makeup group. In addition, it is important to note that the difference between the overall attractiveness ratings across different face sets was not especially large. The mean attractiveness ratings for the faces with no makeup was 3.89 for those in the upper eye condition, 3.39 for the under eye condition, and 3.19 for the lip condition. Thus, although the means were significantly different, the differences were less than 1 point apart and were around center point of the attractiveness scale (3.5). In future studies we will look at these effects more systematically, specifically testing how overall attractiveness might interact with the effects of makeup on attractiveness using our particular stimuli. Also, our overall differences between the faces with and without cosmetics were quite small, but this is consistent with findings in other studies (Jones and Kramer, 2015). Jones and Kramer found that the differences between faces with and without makeup were significant, but smaller than people might predict.

The symmetry analyses of this set of faces show that the addition of makeup sometimes increased symmetry, but at other times it decreased symmetry. These changes in symmetry were consistent with what we predicted would be the most attractive makeup applications, such that upper eye and lip cosmetics led to increased symmetry (potentially increasing attractiveness) and under eye makeup led to decreased symmetry (potentially decreasing attractiveness). Most importantly, the changes in symmetry due to the addition of cosmetics were unrelated to differences in attractiveness ratings. These results suggest that our makeup application did not increase human attractiveness ratings due to a decrease in asymmetry. These results are somewhat surprising, as it seems logical to predict that adding contrast to the eye and lip regions could as a natural consequence make those regions more symmetrical. Even if that is sometimes the case, this change in this case was not big enough to drive the increase in attractiveness we measured.

Facial asymmetry is not only important for attractiveness perceptions, it has also been used in classification algorithms for performing human identification, particularly under external variations caused by expression and illumination changes (Liu et al., 2001, 2003; Mitra and Liu, 2004; Mitra et al., 2006). The results from these studies showed that asymmetry of the face changed very slightly under such factors, making it an effective biometric for recognition. Similarly, cosmetics are another factor that can confound facial recognition methods, thus impairing their accuracies (Dantcheva et al., 2012). Mitra et al. (2018) showed that under such situations, facial asymmetry features are able to perform human identification efficiently. Although these studies show a potentially significant practical application of facial asymmetry in the area of security, further research is required to understand how asymmetry specifically drives recognition methods.

Another limitation of our study is that, in order to increase control, the cosmetics were applied uniformly to all the participants’ faces by an experimenter. It is possible that when a person applies their own makeup they may apply it in a way that significantly decreases asymmetry (whether consciously or unconsciously). However, it is the case that makeup applied to the upper eyes increased attractiveness. Therefore, cosmetics applied this way were still effective in increasing attractiveness, but did not similarly affect facial symmetry. In addition, we did not use color images, in order to limit this exploration to changes in contrast. Many studies have found that color plays an important role in how makeup affects attractiveness (Porcheron et al., 2013; Jones et al., 2015, 2016; Russell et al., 2016), and we plan to explore these effects using these faces in future studies.

In this study we found that increases in contrast affected attractiveness only for the upper eye region, suggesting that the effects reported by Russell (2003, 2009) are primarily driven by the eyes and supporting research showing the importance of eyes in attractiveness perception (Mulhern et al., 2003; Jones et al., 2015). More importantly, we did not find a relationship between human-judged increases of attractiveness based on the addition of cosmetics and a computational model of the symmetry changes for the same faces. Based on these data, we would predict that mechanisms other than symmetry contribute to the increases in facial attractiveness related to cosmetic use in women.

## Ethics Statement

This study protocol was approved by the Institutional Review Committee of California State University Fullerton. Human participants gave written informed consent in accordance with the Declaration of Helsinki.

## Author Contributions

AK and JP conceived of the idea for the behavioral study. SM and JP conceived of the idea for the asymmetry analyses. AK collected the behavioral data. SM designed the asymmetry model and conducted the asymmetry analyses. AK and JP conducted the behavioral analyses. All authors contributed to the writing of this manuscript.

## Conflict of Interest Statement

The authors declare that the research was conducted in the absence of any commercial or financial relationships that could be construed as a potential conflict of interest.

## Footnotes

1 http://illusionoftheyear.com/2009/the-illusion-of-sex/
